# Ingestion of Broccoli Sprouts Does Not Improve Endothelial Function in Humans with Hypertension

**DOI:** 10.1371/journal.pone.0012461

**Published:** 2010-08-27

**Authors:** Buris Christiansen, Natalia Bellostas Muguerza, Atheline Major Petersen, Britt Kveiborg, Christian Rask Madsen, Hermann Thomas, Nikolaj Ihlemann, Jens Christian Sørensen, Lars Køber, Hilmer Sørensen, Christian Torp-Pedersen, Helena Domínguez

**Affiliations:** 1 Department of Oncology, Copenhagen University Hospital, Herlev Hospital, Region Hovedstaden, Denmark; 2 Faculty of Life Sciences, Copenhagen University, Copenhagen, Denmark; 3 Department of Endocrinology, Odense University Hospital, Region Midt, Denmark; 4 Department of Medicine, Sygehus Syd, Region Sjaelland, Denmark; 5 Department of Vascular Biology and Complications, Joslin Diabetes Center, Boston, Massachusetts, United States of America; 6 Department of Cardiology, Copenhagen University Hospital, Gentofte Hospital, Region Hovedstaden, Denmark; 7 Department of Cardiology, Copenhagen University Hospital, Herlev Hospital, Region Hovedstaden, Denmark; University of Sheffield, United Kingdom

## Abstract

Ingestion of glucosinolates has previously been reported to improve endothelial function in spontaneously hypertensive rats, possibly because of an increase in NO availability in the endothelium due to an attenuation of oxidative stress; in our study we tried to see if this also would be the case in humans suffering from essential hypertension.

**Methods:**

40 hypertensive individuals without diabetes and with normal levels of cholesterol were examined. The participants were randomized either to ingest 10 g dried broccoli sprouts, a natural donor of glucosinolates with high *in vitro* antioxidative potential, for a 4 week period or to continue their ordinary diet and act as controls. Blood pressure, endothelial function measured by flow mediated dilation (FMD) and blood samples were obtained from the participants every other week and the content of glucosinolates was measured before and after the study. Measurements were blinded to treatment allocation.

**Results:**

In the interventional group overall FMD increased from 4% to 5.8% in the interventional group whereas in the control group FMD was stable (4% at baseline and 3.9% at the end of the study). The change in FMD in the interventional group was mainly due to a marked change in FMD in two participants while the other participants did not have marked changes in FMD. The observed differences were not statistically significant. Likewise significant changes in blood pressure or blood samples were not detected between or within groups. Diastolic blood pressure stayed essentially unchanged in both groups, while the systolic blood pressure showed a small non significant decrease (9 mm Hg) in the interventional group from a value of 153 mm Hg at start.

**Conclusion:**

Daily ingestion of 10 g dried broccoli sprouts does not improve endothelial function in the presence of hypertension in humans.

**Trial Registration:**

Clinicaltrials.gov NCT00252018

## Introduction

Oxidative stress is considered to play a important role in the processes of cellular injury which ultimately leads to the development of atherosclerosis.[1] However, clinical studies of antioxidants directly administered as a supplement have shown no effects of antioxidants on cardiovascular morbidity and mortality. [2]. An attractive alternative could be to augment the naturally occurring antioxidative defense.[3], [4]

A group of substances which might have this effect are the glucosinolates, substances naturally occurring in cruciferous plants; the glucosinolates are precursors to isothiocyanates, of which sulphoraphane is one of the most potent inducers of the expression of phase-2-enzymes, in mammalian cells.[5], [6], [7].

Among the enzymes induced in vitro the gluthation-S-reductase may be of particular interest since it is instrumental in recycling gluthathion to its reduced state in the cell. In broccoli sprouts younger than 4 days glucoraphanin[5] is abundant and when ingested, glucoraphanin is converted to sulphoraphane. In vitro studies have demonstrated the beneficial effects of glucosinolates on mammalian cell lines[7]. Wu and colleagues demonstrated that daily treatment of Stroke Prone Hypertensive rats with dried broccoli sprouts after weaning attenuated the development of hypertension and improved endothelial function of the carotid arteries.[8]

We therefore decided to study whether the administration of dried broccoli sprouts in patients with essential hypertension could decrease blood pressure and increase flow mediated vasodilation(FMD). We have focused on endothelial function since this parameter previously has been demonstrated to be influenced by the level of oxidative stress, and because it is a sensitive predictor for oxidative stress as well as for the future development of atherosclerosis.[9], [10]

We hypothesized that an increase in flow mediated vasodilation shooud be detectable in the interventonal group after four weeks of treatment both if we used the participants as their own controls and if we compared them to a control group. This was our primary outcome.

Secondary outcomes in our study were measurement of blood pressure and lipoproteins where we were interested in seeing if the blood pressure would decrease during treatment and if lipoproteins would show an increase in HDL as well as a decrease in LDL.

## Methods

The protocol for this trial and supporting CONSORT checklist are available as supporting information; see Checklist S1 and Protocol S1.

### Participants

Volunteers were enrolled in the study after advertising in local newspapers for non smoking hypertensive individuals without known diabetes or hypercholesterolemia. In- and Exclusion criteria are listed in Table 1.

**Table 1:** In and exclusion criteria for the participants.

**Inclusion Criteria**
• Hypertension, defined as a diastolic blood pressure of above 90 mm Hg or a systolic blood pressure of more than 140 mm Hg or in medical treatment for hypertension.
**Exclusion Criteria**
• Impaired glucose tolerance, measured by an oral glucose tolerance test or diabetes according to internationally recognized criteria
• Known hypertension due to renal artery stenosis
• Hypercholesterolemia defined as a total cholesterol of more than 5 mM or statin treatment,
• Smokers
• Patients receiving vitamin K-antagonists (coumarine, marcoumar)
• Women of childbearing age with no safe method of contraception
• Pregnant or breastfeeding women
• Patients under the age of 18 at inclusion in the study

### Ethics

The study was approved of the ethics committee for Copenhagen and Frederiksberg municipalities (no 01-257/04). Written informed consent was obtained from all participants.

### Intervention

Endothelial function was measured on each individual three times, at start of treatment and twice during the four weeks of treatment with an interval of two weeks. Blood samples for erythrocyte content and inflammation, samples for lipids, blood glucose and inflammation were taken at each visit. Furthermore samples were obtained for analyzing the blood content of glucosinolates and their metabolites. The purpose for measuring the erythrocyte content blood glucose and inflammatory status was to insure that the stimulus for measuring FMD was as uniform as possible for each visit since a change in blood viscosity could influence the shear stress of the vessel wall and therefore increase the measured FMD, likewise elevated blood glucose has been showed to decrease FMD response. Endothelial function was measured using Flow Mediated Dilation (FMD) using a protocol following current guidelines.[11], [12] The participants were instructed to arrive for the investigation after fasting for the last 12 hours including medication. After arrival they were placed in supine position for at least 10 minutes before initiation of measurements. The examination room was quiet and had a temperature of about 22°C and had subdued lighting. Measurements were performed on the brachial artery of the right upper arm approximately 10 cm above the antecubital fossae with the pneumatic tourniquet placed distally to the ultrasound transducer immediately below the antecubital fossae at the area of greatest width of the forearm. The tourniquet was inflated to a pressure of 300 mm Hg for a period of 4 minutes and 45 seconds. During the last 15 seconds of inflation and the first 15 seconds after deflation of the tourniquet, a Doppler spectroscopy of the flow through the brachial artery was recorded, permitting calculation of the flow increase through the brachial artery. As a control, the flow independent dilation (FID) was measured 10 minutes after the measurement of FMD by administering 100 µg nitroglycerin sublingually. Measurement of FMD and FID were given as the relative increase in diameter in percent compared to the baseline diameter of the brachial artery. In order to asses the possible changes of shear stress during the study, blood samples were analyzed for the relative content of red blood cells and inflammatory markers (leukocytes and c-reactive protein) since these factors usually are the most important contributors to the viscosity of the blood.[13] The measurements were performed on an Acuson XP10/4 ultrasound apparatus using a 7 MHz linear transducer (L7), and the images were analyzed real time on a Personal computer using a NI PCI-1470 frame grabber and Vascular Image Acquisition (VIA) software.[14], [15] The visits were made on the same time of the day in order to avoid diurnal variation of FMD.

### Procurement of sprouts

The broccoli sprouts were obtained from a commercial grower (Van Der Plass Sprouts BV, the Netherlands) and were sprouted for 3 days. Immediately after harvesting they were refrigerated and subsequently dried at 40°C.The dried sprouts were afterwards packaged in airtight plastic bags each containing 10 grams of dried sprouts, equivalent to 100 g fresh sprouts. The dried sprouts had the glucosinolate content measured using previously described methods[16], [17]. In accordance with the study by Wu et al. [8] it was attempted to construct a placebo for the control group by freezing/thawing sprouts before drying. However this preparation still contained large amounts of glucosinolate. Therefore the study was conducted without placebo treatment of the control group, which served as time-control. Before the start of the study and after the analysis of the glucosinolate content we did consider if it would be better to use another type of sprouts for the control group in order to keep participants as well as investigators blinded, We decided against this because we were not able to provide such a placebo with taste and smell as the broccoli sprouts, and because we were not able to get a placebo which would be identical to the broccoli sprouts in all aspects but the glucosinolate content.

### Randomization and design

Patients were randomised to treatment with broccoli sprouts or control in a 1∶1 ratio. Treatment consisted in a daily ingestion of 10 g dried broccoli sprouts. The block size for randomization was 4. Randomization was performed by an assistant in sealed envelopes and group allocation was blinded to the investigator. The participants were thus unblinded with regards to their allocation but the investigator was blinded throughout the study to the status of the participants. Since broccoli sprouts are not commercially available and have a much larger content of glucoraphanin than mature broccoli, we were confident that the control group would not ingest glucoraphanin in substantial amounts during the study. Pre-study antihypertensive medications were maintained during the study for all participants. The participants were given a package containing 28 bags with 10 g dried broccoli sprouts in each, instructions were to eat the content with one of the meals of the day, they were also instructed to avoid eating or drinking for at least 8 hours prior to each day of study. As part of our efforts to determine compliance we took blood samples from the participants at each session and the participants were informed that this also was done in order to estimate the content of the active substances in their blood stream. The investigator was blinded during the study and the preliminary statistical analys, During the study the analysis of blood samples and measurement of FMD was done immediately after each session and the data were entered into the study database. The investigator was unaware of the allocation of the participants during the study. After conclusion of the study the allocation of each participant into two groups was given the investigator, but during this part it was still not revealed which group had been the control and which had been the interventional group.

### Objectives and Outcomes

Following the hypothesis outlined in the introduction our primary outcome was if we would observe an increase in FMD in the interventional group during the intervention.Secondary outcomes were changes in blood pressure and changes in levels of blood cholesterol during the study in the interventional group.

### Statistical Methods

The results were analyzed using SAS software (version 9.10). Comparisons between intervention and control group were analysed by non-paired Student's t-test and comparison of changes over time for the intervention and the control group were performed using a paired students test. Results were presented with calculated mean values and confidence intervals for the measurements at the different session as well as for the differences between values in the two groups.

It was assumed that a detectable effect would produce at least a 3% difference in FMD, on this assumption it was calculated that our study would have a power of 90% with 20 individuals in each group.

## Results

### Participant flow

**Figure 1:**
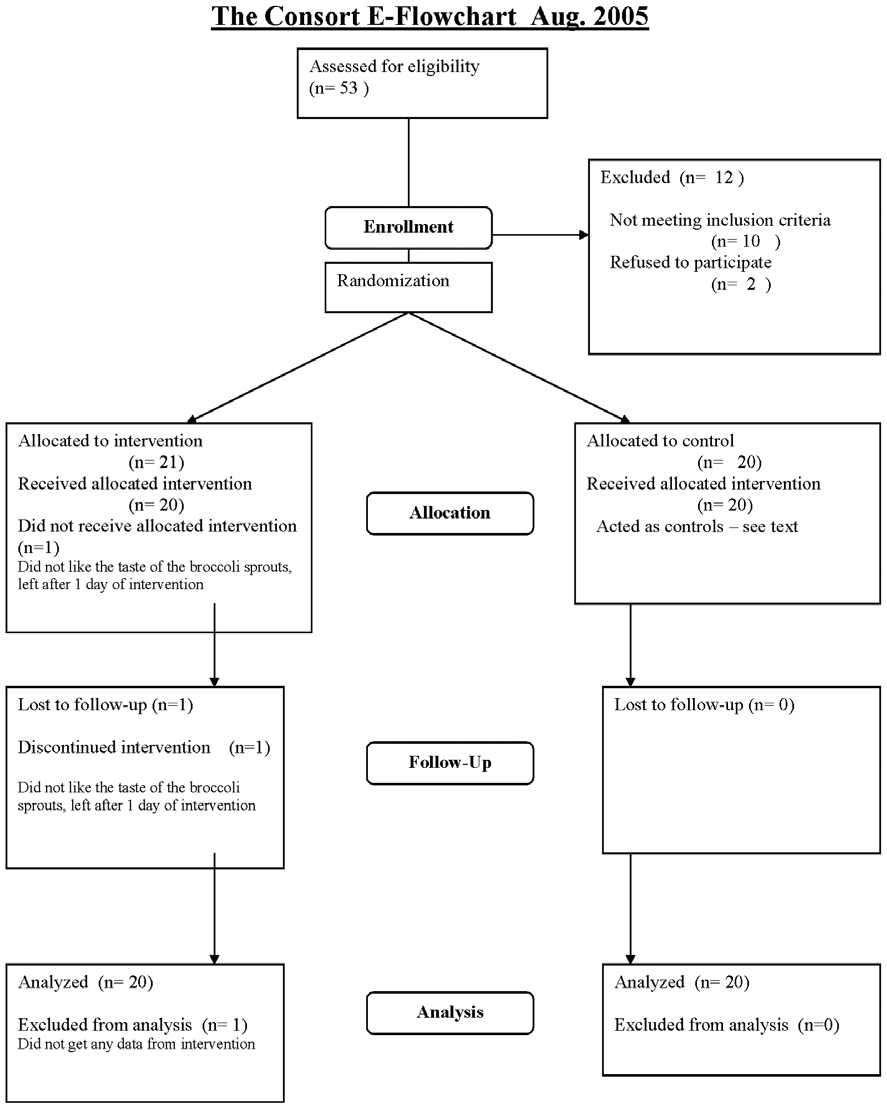
Consort 2001 flow diagram.

The study enrolled 41 participants, 20 in the control group and 21 in the interventional group. In all 53 persons were screened prior to enrollment, of these10 did not meet the enrollment criteria and 2 decided not to participate. The reason for including one more in the interventional group was that one participant dropped out one day after receiving the sprouts owing to the disagreeable taste of the sprouts. One participant in the interventional group missed one visit because of hospitalization (leg fracture). All other participants received treatment as allocated and participated in examinations as planned. The baseline characteristics for the participants are listed in Table 2 and the participant flow diagram is presented in Fig. 1.

**Table 2:** Baseline characteristics of the control and interventional group.

	Control	Sd	Range	Intervention	SD	Range	p
Male	5	n/a	n/a	11		n/a	0.10
Female	15	n/a	n/a	10		n/a	0.50
Age	54	10	32–67	58	9	38–77	0.23
Cigarettes(Packet years)	6.9	17	0–70	6	10.4	0–41	0.84
BMI	26.2	3.2	22–33.7	29.1	6.6	21–51	0.08
Systolic mean	158.6	20.4	132–215	158.5	20.9	121–196	0.98
Diastolic mean	98	7.8	88–115	96	10.4	80–115	0.50
Plasma Glucose	5.3	0.4	4.5–5.9	5.4	0.4	4.5–6.4	0.40
total Cholesterol	5	0.7	3.6–7.1	5.1	1	3.6–7.6	0.55
HDL	1.7	0.5	.9–2.8	1.7	0.6	1.1–3.4	0.85
LDL	2.9	1	2–4.2	3	0.8	1.7–5.1	0.32
Triglycerides	1.09	0.7	0.43–3.59	1.03	0.6	0.38–2.5	0.71
Antihypertensive drugs	users			users			p
C03 Diuretics	4	n/a	n/a	5	n/a	n/a	0.77
C07 β-Blockers	2	n/a	n/a	2	n/a	n/a	0.96
C08 Calciumantagonists	7	n/a	n/a	3	n/a	n/a	0.12
C09 ACE inhibitors or ATII antagonists	8	n/a	n/a	12	n/a	n/a	0.27

Since all participants were non smokers at the time of the investigation, packet-years refers to years of smoking prior to the study. Antihypertensive drugs are grouped by ATC.

### Vascular measurements

**Figure 2:**
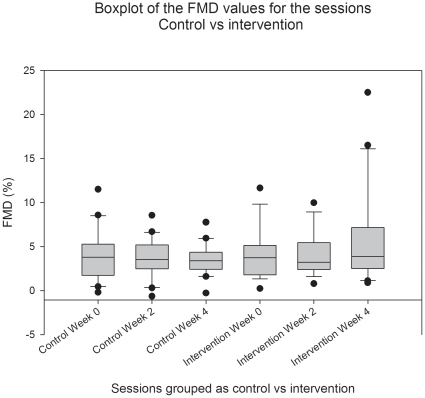
Box plot of the FMD values for weeks 0, 2 and 4 during the study for the Control and Interventional group.

**Figure 3:**
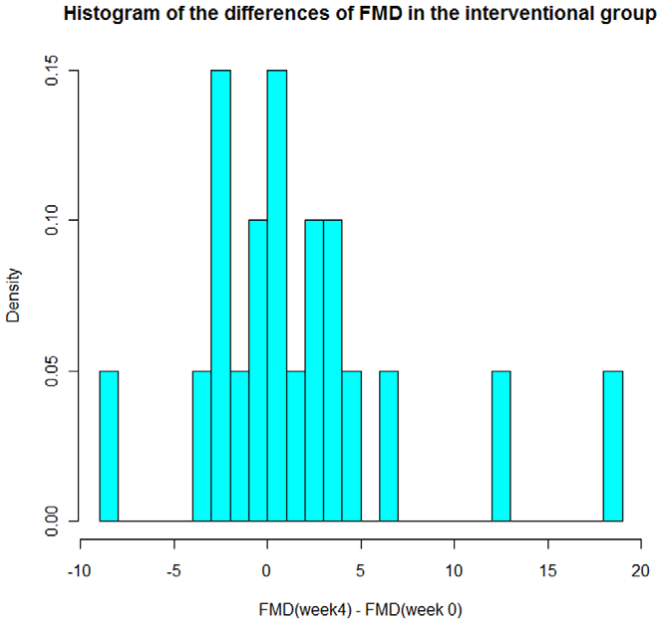
Histogram of the differences in FMD between week 4 and week 0 in the interventional group.

The FMD values with 95% confidence interval and p values calculated using students test for the intervention and control group are displayed in Table 3; overall, no significant changes occurred in the two groups during the period of treatment. The change in mean FMD between weeks 0 and 4 is greater by 2.25% in the intervention group compared with the control group. The 95% confidence interval for this difference between changes is (−0.73%; 5.23%). FMD was slightly lower in the control groupas seen by Fig. 2, and the average vascular diameter was slightly lower in the control group (data not shown). Figure 2 shows boxplots of the FMD values in the interventional as well as the control group, of notice is that the median values for the FMD in either group did not change during the study. In order to visualize the causes for the observed changes in the interventional group for week 0 to 4, we have in Figure 3 plotted a histogram for the distribution of changes in the FMD values in the interventional group between week 0 and week 4.

**Table 3:** Development of FMD in percent during the study.

	Week	0	2	4	Change 0–4
Intervention	Mean	4.07	4.12	5.80	1.78
	CI	2.75 to 5.39	2.93 to 5.31	3.19 to 8.41	−0.95 to 4.41
Control	Mean	4.08	3.60	3.56	−0.52
	CI	2.70 to 5.46	2.55 to 4.64	2.74 to 4.38	−1.99 to 0.94
Difference	Mean	−0.01	0.53	2.24	2.25
	CI	−1.88 to 1.84	−1.01 to 2.06	−0.46 to 4.98	−0.73 to 5.23

Mean and confidence interval (2,5–97,5%) none of the differences were significant on the 0.05 level using Students Test.

### Blood Samples

For the blood test taken simultaneously with the FMD measurements, we have not been able to detect any differences in these samples in the course of our study. The results for are shown in Table 2 and 4 We present the values at at randomization, as baseline values. The variation we observe in these blood samples appeared basically to be of a random nature and we were not able to demonstrate any systematic change during the experiment.

**Table 4:** Mean, confidence intervals and p-values for selected blood samples, p-values calculated using Students test.

Total Cholesterol		Intervention			Control		
Weeks	mean	CI 2.5	CI 97.5	mean	CI 2.5	CI 97.5	p-value
0	5.08	4.62	5.53	4.95	4.65	5.24	0.62
2	5.07	4.57	5.57	4.88	4.62	5.14	0.49
4	5.04	4.55	5.53	5.06	4.76	5.36	0.94
HDL							
Weeks	mean	CI 2.5	CI 97.5	mean	CI 2.5	CI 97.5	p-value
0	1.73	1.49	1.97	1.81	1.54	2.07	0.67
2	1.67	1.40	1.94	1.73	1.50	1.96	0.72
4	1.65	1.40	1.90	1.78	1.54	2.01	0.45
LDL							
Weeks	mean	CI 2.5	CI 97.5	mean	CI 2.5	CI 97.5	p-value
0	2.76	2.29	3.22	2.78	2.51	3.04	0.94
2	2.72	2.26	3.19	2.72	2.48	2.95	0.98
4	2.89	2.49	3.28	2.80	2.53	3.06	0.70
Haematochrite							
Week	mean	CI 2.5	CI 97.5	mean	CI 2.5	CI 97.5	p-value
0	0.39	0.34	0.43	0.58	0.20	0.96	0.31
2	0.41	0.39	0.42	0.40	0.39	0.41	0.17
4	0.40	0.39	0.42	0.39	0.38	0.40	0.24

### Development in blood pressure

Systolic, diastolic as well as mean blood pressure did not change significantly during the study. Differences detected in the study were related to gender and age.

Results are shown in Tables 5 and 6.

**Table 5:** Development of Systolic Blood Pressure during the study.

		0	2	4	Change 0–4
Intervention	Mean	152.30	149.21	145.15	−7.80
	CI	143.10 to 161.50	141.45 to 156.97	136.57 to 153.73	−19.13 to 3.53
Control	Mean	145.85	148.75	144.50	−0.70
	CI	136.93 to 154.77	139.17 to 158.33	137.58 to 151.42	−14.44 to 13.04
Difference	Mean	6.45	0.46	0.65	−5.80
	CI	−5.94 to 18.84	−11.48 to 12.40	−11.33 to 10.03	−24.34 to 10.14

Mean and confidence interval (2,5–97,5%) none of the differences were significant on the 0.05 level using Students Test.

**Table 6:** Development of Diastolic Blood Pressure during the study.

		0	2	4	Change 0–4
Intervention	Mean	89.70	90.58	88.65	−1.05
	CI	83.73 to 95.66	84.88 to 96.28	84.04 to 93.26	−8.18 to 6.08
Control	Mean	91.80	89.55	88.75	−3.05
	CI	86.53 to 97.07	85.32 to 93.78	84.84 to 92.66	−9.12 to 3.02
Difference	Mean	−2.10	1.03	−0.10	2.00
	CI	−9.80 to 5.60	−5.80 to 7.91	−5.95 to 5.75	−7.06 to 11.06

Mean and confidence interval (2,5–97,5%) none of the differences were significant on the 0.05 level using Students Test.

### Glucosinolate content of the broccoli sprouts

The glucosinolate content of the sprouts was determined before and after the drying and packaging had taken place in order to ensure that the content of glucosinolates in the dried sprouts was known and to asses the possible deterioration of the glucosinolates due to the drying.

Measurements of the concentration in the sprouts at the end of the study were similar to those prior to the study (Table 7). The sprouts had a glucoraphanin content of 25.9±8.5 µmol/g dry weight and a total glucosinolate content 48.5±14.2 µmol/g dry weight.

**Table 7:** Concentration of glucosinolates in the sprouts prior to and after the study.

	Prior to study			After study		
	Mean (μmol/g)	D	proportion	Mean (µmol/g)	SD	proportion
Glucoraphanin	30.3	4.0	75.0%	25.9	8.5	53.4%
Total	40.4	5.8	100.0%	48.5	14.2	100.0%

All concentrations are given as µmol/g with the proportion signifying how large a proportion of the glucosinolate content consisted of Glucoraphanin.

As stated in the methods section blood samples were drawn at each visit in order to determine the glucosinolate content in the blood of the participants. Analysis of these samples did not show detectable levels of glucosinolates or isothiocyantaes in any of the participants.

### Adverse events

Apart from one participant leaving the study because of the taste of the sprouts which made her very uncomfortable, no adverse effects were observed during the study.

## Discussion

In the current study we have tested whether dietary glucosinolates can improve endothelial function in patients with hypertension as it has been demonstrated in animal models.[8] Using supplements of broccoli sprouts with preserved concentrations of the glucosinolate glucoraphanin, we found no significant change in endothelial function, measured twice during an investigator-blinded 4-week treatment period. We are aware of the fact that a change does take place in the interventional group between week 0 and week 4 even if this change is insignificant. An issue could therefore be if this lack of significance was due to insufficient power of the study. We find this unlikely, since, as demonstrated in Figure 3 it is very few of the participants in the interventional group who actually increase their peak dilation during the study. At Figure 3 it can also be seen, that the majority of the observed changes cluster around 0 with 75% having a change in FMD of less than 3%, our stated minimum observable difference. If we look at the distribution of the absolute difference in peak diameter for week 0 and 4, we find a similar pattern with 75% having a peak diameter difference of 0.26 mm or less, values which are very close to the measuring uncertainty of the method. As Figure 3 shows the mean increase in FMD and peak diameter of week four is mainly due to two participants, who have very pronounced increases in their response. Furthermore, the confidence interval (−0.73− 5.23) quoted in the results section indicates that broccoli sprouts have an effect of at most 5% on the mean FMD. As such we find it justified to state, that we do not observe an increase in FMD in our study group.

During this period, we have not been able to demonstrate any change in blood pressure, or blood samples reflecting blood lipids or inflammatory status. This finding is at odds with experiments carried out on spontaneously hypertensive stroke prone rats (SHSP), which may be due to a variety of factors. The animal study was designed to investigate if a daily dose of sprouts would protect against the development of hypertension, while we only studied endothelial function in patients with established hypertension. Furthermore, dosage as well as duration of treatment was also different, as the rats were given dried sprouts from weaning and until termination of the experiment after fourteen weeks, which translated to a human lifespan would imply treatment form early childhood into adolescence. Finally, hypertension is not a well described disease entity in humans whereas the SHSP are a distinct breed of rats characterized by their tendency to develop hypertension and stroke.[18], [19] It is therefore very likely that the rats are much more uniform in the genetic layout which determines their tendency to hypertension and in the mechanism by which they develop their hypertension. In contrast the genes determining hypertension in humans are probably heterogeneous. It is therefore possible that the mechanism we tried to influence is not the relevant mechanism in all humans. For cancer, epidemiological studies suggest that in humans, the possible beneficial effects of brassica in the diet is dependent on genetic polymorphisms in the target population[20].

Our study has some limitations that need to be addressed. The dose we gave was, if related to body mass, approximately 7 times smaller than the dosage given to the rats in the study discussed above[8], however prior to the study we tried to establish a dose which would be tolerable to humans and decided against a larger dose, since it was associated with gastrointestinal side effects. Our dose is also comparable with doses given to humans in other studies, which have demonstrated an effect of broccoli sprouts on humans in a similar timespan.[21], [22], [23], [24], [25]

The fact that we were not able to detect glucosinolates in the blood of the participants raises the question if lack of compliance could be a reason for the lack of observed effect. Other studies does indicate that the glucosinolates in the precense of myrosinase will be transformed to isothocyanates at an early stage of digestion[26] so this does not necessarily mean that our participants were non compliers. However, judging from the overall behavior of our participants and due to the fact that some of the participants in the interventional group wrote to us to tell how much they had liked the taste of the sprouts, we do not believe this has been the fact. Furthermore during the study the participants were informed of our intention of analyzing for glucosinolates in their blood samples. Another question which could be raised is whether the observed FMD in the two groups is impaired or not. Indeed, the FMD measured in the individuals in this study (total average 4.2±3.2) is lower than the FMD values we have observed in groups with healthy non smoking volunteers (5.7±2.4). Since other studies have presented effect on FMD after treatment with drugs or other substances in a comparable time-span to the 4 weeks used in our study and since an increase in gene expression after exposure of broccoli has been observed in the human intestine 6 hours after ingestion[21], it should be expected to see an effect of glucosinolates on endothelial function after four weeks of treatment.

As stated in the result section we did not find any changes in blood lipids measured during the intervention period. This is at odds with other experiments carried out in humans and with similar doses of sprouts, since Murashima et al demonstrated a decrease in total and LDL cholesterol in humans after one week of treatment with 50 g of fresh broccoli sprouts given daily, our daily dose had twice the glucosinolate content, comparable to 100 g fresh sprouts. We used dried broccoli sprouts as opposed to fresh sprouts, since other studies have shown a rather fast deterioration of the glucosinolate content in fresh broccoli[27], [28]. Our study had a longer duration of treatment than the study by Murashima et al., which suggests that the effect of glucosinolates on lipids may be an early transient one or that other substances in fresh sprouts can be attributable to the changes observed in the study of Murahisma et al,[24] We therefore conclude that a four week treatment of dried broccoli sprouts, given as daily doses of 10 g does not improve endothelial function in patients with established hypertension, and does not change serum cholesterol levels. Further investigations are needed to elucidate if broccoli sprouts or glucosinolates have a positive effect on endothelial function in subgroups of hypertensive patients or other patient groups.

## Supporting Information

Checklist S1 CONSORT Checklist.
(0.19 MB DOC)

Protocol S1 Trial Protocol, in Danish.
(0.10 MB DOC)
