# Supplementary material for: Ingestion of Broccoli Sprouts Does Not Improve Endothelial Function in Humans with Hypertension
Source: PLoS One. 2010 Aug 27;5(8):e12461. doi: 10.1371/journal.pone.0012461 (PMC2929197; doi:10.1371/journal.pone.0012461)
Supplement: Protocol S1 — Trial Protocol, in Danish. (0.10 MB DOC) [file pone.0012461.s002.doc]

## Kan broccolispirer beskytte mod udvikling af åreforkalkning?

## Forskningsprotokol

## Virkning af Broccolispirer som kosttilskud på vaskulær endothelfunktion hos personer med risiko for udvikling af åreforkalkningssygdom

### Baggrund

Ved oxidativt stress forstås en ubalance af produktion af frie iltradikaler og andre oxidanter i forhold til kapaciteten af antioxidanter i organismen som potentielt kan lede til vævsskade[1]. Oxidativt stress i blodkar udgør et led i patogenesen ved åreforkalkningssygdom. Der er påvist tegn på vaskulært oxidativt stress ved mange risikotilstande for åreforkalkningssygdom, blandt andet hyperkolesterolæmi, hypertension og diabetes[2]. Ved disse tilstande kan der lang tid før manifest åreforkalkning måles nedsat endotel-afhængig vasodilatation, blandt andet fordi frie iltradikaler nedbryder nitrogenoxid (NO).

Trods overvældende evidens for, at oxidativt stress har betydning for åreforkalkningssygdom i epidemiologiske og mekanistiske studier har de fleste kliniske interventionsstudier ikke kunnet påvise at antioxidanter, for eksempel antioxidante vitaminer, kan forebygge kardiovaskulære begivenheder[1, 2]. Årsagen hertil er måske at langtidsadministration af antioxidante vitaminer ikke sikrer tilstrækkelige koncentrationer i det relevante compartment(vaskulært, inter- eller intracellulært)[2], eller at administrationen af antioxidanter ikke har været effektive i forhold til de oxidanter der er tale om [1].

Derfor kan en bedre strategi for antioxidant behandling bestå i at øge organismens egen antioxidante forsvar, enten ved øget endogen produktion af antioxidanter eller ved øgning af de reparative processer i organismen.

Planter tilhørende kålfamilien (Brassicae) indeholder stoffer kaldet glucosinolater som under tilberedelse og fordøjelsen metaboliseres til isothiocyanater, der har geninducerende egenskaber i den menneskelige organisme, og som derved  *in vitro*  øger cellers reparative processer og inducerer dannelse af antioxidante enzymer.

Disse planter har endvidere i epidemiologiske studier vist sig at have cancerforebygende effekt.[3, 4]

Det er vist at broccoli-spirer indeholder markant store mængder af glucoraphanin og at dette i organismen metaboliseres til sulphoraphane, som inducerer ekspressionen af antioxidante enzymer *in vitro* [4, 5]

Studier af cancerudvikling har også vakt interesse for om induktion af antioxidante enzymer har en særligt beskyttende effekt mod cancer [3, 4] Det er således vist at tilstedeværelse af disse stoffer er associeret til mindre cancer forekomst [3], andre undersøgelser har vist at kosttilskud af broccoli-spirer beskytter mod cancer i en dyremodel[4]

Et nyligt studie har vist at 2 ugers administration af broccoli-spirer i kosten hos en rottemodel for hypertension reducerer markører for oxidativt stress, bedrer vaskulær endotelfunktion og sænker blodtrykket[6].

### Hypotese

Indholdsstoffer i broccoli-spirer, herunder glucoraphanin der metabolisres til sulphoraphane, inducerer gener for antioxidante enzymer. Derved kan tilskud af broccoli-spirer i kosten bedre funktionen af det vaskulære endotel hos personer med risiko for åreforkalkningssygdom, som kan måles ved en bedring af flow medieret vasodilatation.

### Formål

At undersøge om 8 ugers behandling med broccoli-spirer som kosttilskud kan bedre flow-medieret vasodilatation hos patienter med hypertension, hyperkolesterolæmi og diabetes.

Samt: at undersøge hvilke stoffer fra broccolispirerne samt disses metabolitter, der kan genfindes i organismen.

### Design

Randomiseret, placebokontrolleret, dobbelt-blind undersøgelse.

### Forsøgspersoner

Forsøget vil inkludere patienter med med risiko for udvikling af hjertekarsygdomme herunder hyperkolesterolæmi, hypertension eller diabetes forsøgspersonerne randomiseres til henholdsvis behandling eller placebokontrol. Derudover inkluderes der 40 raske forsøgspersoner , som udvælges således at de er matchet med patienternes alder, køn og body mass index (BMI).

I alt vil der indgå 160 forsøgspersoner.

Patienter rekrutteres fra Kardiologisk Afdeling Y, Bispebjerg Hospital. Raske forsøgspersoner rekrutteres efter annoncering i lokalavis.

Inklusionskriterier for patienter :

LDL-kolesterol > 3.5 mM med eller uden behandling med kolesterolsænkende medicin *eller*

Systolisk blodtryk (konsultationsblodtryk) > 150 mmHg *eller*

Kendt type 2 diabetes med varighed > 3 måneder og HbA_1c_ > 9%.

Eksklusionskriterier for patienter:

Alder < 18 år

Graviditet

Behandling med Marevan eller Marcoumar (K-vitamin antagonister)

Rygere

Eksklusionskriterier for raske forsøgspersoner:

Tilstedeværelse af de ovenstående eksklusionskriterier for patienter

Systemisk akut eller kronisk sygdom

LDL-kolesterol > 2,5 mM

Systolisk blodtryk > 120 mmHg

Fastende kapillær blodglukose > 5,2 mM

Kvindelige forsøgsdeltagere i fertil alder, vil før inkludering i projektet blive udspurgt om dato for sidste menstruation og få foretaget seHCG (graviditetstest), de vil ikke blive inkluderet i projektet hvis denne er positiv. De vil samtidig blive bedt om at være opmærksom på om næstkommende menstruationer kommer på forventet tidspunkt.

Ved udeblivelse af menstruation med mere end tre dage fra det forventede tidspunkt, vil ny seHCG blive taget. Er se HCG negativ, men menstruation fortsat udebliver, vil ny se HCG blive taget efter endnu en uge.

Ved positiv seHCG vil forsøgsdeltageren udgå af projektet.

### Statistisk styrke

Baseret på variabiliteten af undersøgelser i vores laboratorium forudsættes en standardvariation for forskellen på 0,8% (idet flow-medieret vasodilatation er et mål for relativ dilatation uden enhed, angivet i %). Det fastsatte antal patienter i hver gruppe giver en statistisk styrke på 90% for at påvise en ændring af af flow-medieret vasodilatation på mindst 30% med en sandsynlighed på 0,05 for type I fejl.

### Randomisering og blænding

Randomisering af patienter til enten en behandlingsgruppe eller en placebokontrolgruppe vil foregå ved at en uafhængig medarbejder forbereder konvolutter med randomiseringskode baseret på computer-genererede tal. Konvolutterne åbnes i forudbestemt rækkefølge når en forsøgsperson inkluderes i studiet..

### Kosttilskud

Broccoli-spirer vil blive dyrket i vækstmediefri betingelser og under hygiejnisk kontrol i maltningsanlæg. Spirerne høstes 4 dage gamle

Halvdelen af spirerne tørres,under betingelser som skulle optimere for indhold af glucoraphanin og dettes aktive metabolit sulphoraphan[7]

Kontrol-kosttilskud vil består af broccoli-spirer som er frosset, tøet og efterfølgende tørret , fryse-tø proceduren nedbryder cellevæggen og medfører metabolisering af glucoraphanin til sulphoraphane nitril der ikke har enzyminducerende aktivitet i organismen[7].

Indholdet af sulphoraphan og sulphoraphan nitril i prøverne vil blive verificeret ved metoder som tidligere beskrevet [7, 8]

De tørrede spirer vil blive pakket i dagsdoser af 100 g og mærket med kode, så hverken lægen som udleverer kosttilskuddet eller forsøgspersonen véd om det er det virksomme kosttilskud eller placebo-kosttilskuddet.

Behandlingen gives i 8 uger.

### Blodprøver og urinprøver

Total kolesterol, LDL-kolesterol, HDL-kolesterol, triglycerid, blod-glukose, CRP og HbA_1c_ vil blive målt i blodprøver taget i fastende tilstand før inklusion, ved måling af flow medieret vasodilatation og ved behandlingsperiodens afslutning. For kvindelige forsøgsdeltagere vi der desuden blive målt seHCG som ovenfor anført under inklusionskriterier.

Sulphoraphane og eventuelt andre indholdsstoffer af broccoli-spirer eller deres metabolitter vil blive målt i blodprøver og i urinprøver taget før inklusion og med intervaller af 2 uger i behandlingsperioden samt ved behandlingsperiodens afslutning.

Målingerne af indholdsstoffer og disses metabolitter vil blive udført af en gruppe uafhængigt af den kliniske gruppe, på den Kongelige Veterinær og Landbohøjskole og disse målinger kan danne baggrund for en selvstændig publikation fra denne gruppe, beskrivende optag og metabolisering af indholdsstoffer af broccolispirer i den menneskelige organisme.

Totalt forventes udtaget en mængde blod svarende til 15 ml ved hver undersøgelse.

### Flow-medieret vasodilatation

Endotelafhængig vasodilatation vurderes ved at måle udvidelsen af a. brachialis efter kort tids afklemning af blodtilførslen til underarmen.

Som kontrol anvendes måling af endotheluafhængig vasodilatation efter sublingual administration af nitroglycerin (glycerylnitrat).

Denne teknik er særdeles velbeskrevet[9].

Desuden er den veletableret i vores laboratorium, hvor vi har udført over 100 undersøgelser de seneste 2 år.

Målingen foretages medens forsøgspersonen ligger i hvile, på den ene arm fremstilles et tværsnitsbillede af a.brachialis med ultralyd.

Ved passende metode sikres, at man kan identificere slutdiastolen i a. brachialis og der foretages registrering af følgende sekvenser af a.brachialis.

- Før armen afklemmes, her registreres dels længdsnit af a.brachialis for bestemmelse af diameter i slutdiastole, dels foretages en registrering af flow gennem a.brachialis ved hjælp af ultralydsdoppler.
- Efter afklemning af underarm med pneumatisk tourniquet oppustet til 300 mmHg over 5 min og efterfølgende retablering af flow til underarmen. Også her registreres dels flow ved ultralydsdoppler som ovenfor anført, dels registreres diameter af a.brachialis i slutdiastole i et inverval varende op til 2 minutter efter at afklemningen er ophørt. Normalt vil man i denne fase observere en dilatation af a.brachialis som udtryk for at karrets endothel frigiver NO som reaktion på det øgede flow og dermed opståede shear-stress. Ved oxidativt stress ses en mindre kardilatation som følge af, der ikke bliver dannet samme mængde NO i karvæggen.
- Som kontrol af forsøgspersonens evne til kardilatation vil anvendes NO uafhængig kardilatation, dette fåes ved at registrere kardiameteren som ovenfor anført, og registrere den i 5 min efter indgift af Nitroglycerin.

De herved fremkomne længdesnit optagelser af a. Brachialis vil blive behandlet ved hjælp af validerede målemetoder til bestemmelse af dilatationen af a. brachialis .[9] Dilatationen vil blive bestemt som forholdet af diameteren af A. Brachialis i slutdiastole før oppustning af tourniquet i forhold til maksimal dilatation i slutdiastole efter at trykket atter er taget af tourniqueten, henholdsvis efter Nitroglycerin indgift.

De fremkomne værdier opgives som relative ændringer i kardiametren, typisk i procent.

Maksimal endothelafhængig dilatation forekommer normalt i peridoden 45-90 s efter fjernelse af trykket.

Endotheluafhængig vasodilatation efter indgift af Nitroclycerin sublingualt forekommer normalt ca. 3 min efter indgift af nitroglycerin.

### Etiske overvejelser

Projektet vil blive udført i overensstemmelse med Helsinki Deklaration II. Forsøgspersonerne vil blive informeret skriftligt og mundtlig om forsøget. De vil før deltagelse med deres underskrift bekræfte, at de har forstået informationen og at de ønsker at deltage (se *Deltagerinformation og samtykke-erklæring*). Der vil blive givet økonomisk kompensation til forsøgspersonerne for deres deltagelse i forsøget.

Der er foreligger oplysninger om skadelige virkninger ved indtagelse af broccoli-spirer, hverken ved menneske eller dyreforsøg

I USA og Canada sælges broccolifrø til private med henblik på spiring som kosttilskud.

Tidligere undersøgelser har antydet at der skulle kunne være en toksisk effekt af indol og β-hydroxyalkenyl glucosinolater, disse glucosinolatgrupper er til stede i næsten umålelige koncentrationer i broccolispirer.[10]

Indtag af planter af brassica familien i øvrigt har i flere undersøgelser vist at have en cancerbeskyttende effekt, hvorfor man i dag generelt anbefaler et øget indtag af disse planter. [3, 5, 11]

Ultralydsundersøgelse af flow-medieret vasodilatation er i sig selv ufarligt og kun forbundet med let ubehag.

Administration af nitroglycerin er særdeles sikkert, men kan afstedkomme hovedpine, rødme af huden, svimmelhed, kvalme, og blodtryksfald. Disse bivirkninger forsvinder som regel efter 10-30 minutter. Forsøgspersonen vil 30 minutter efter administration af nitroglycerin hvile i liggende stilling og være konstant overvåget.

En mere detaljeret fremstilling af de etiske overvejelser vedrørende forsøget er vedlagt som bilag til ansøgningen til den Videnskabsetiske komite.

*Information og Samtykke*

Skriftlig information, foreligger som informationsbrev til forsøgsdeltagerne

(se bilag1).

Proceduren for informeret samtykke vil have to arme, idet selve rekrutteringen af deltagere til projektet forestilles foretaget på to måder, dels ved kontakt med patienter indlagt på kardiologisk afdeling Y12 ogY21 ved udskrivning herfra, dels ved annoncering i dagspressen (annoncetekst vedlagt ansøgningen som bilag).

Rekruttering fra afdeling:

På de to involverede afdelinger vil der blive afholdt orienteringsmøde for det ansatte personale, således at de er i stand til at informere patienter som kunne indgå i studiet om dettes eksistens og formål.

I fald patienterne er interesserede, vil en af medlemmerne fra gruppen komme op og orientere dem om projektet mundtligt samt give dem de relevante skriftlige orienteringer incl. pjecen ”Før du bestemmer dig”.

Der vil derefter blive aftalt en tid, hvor patienten kan kontaktes med henblik på en undersøgelse, hvor patientens samtykke endeligt kan blive indhentet og hvor det kan sikres at patienten har læst og forstået det skriftlige forsøgsmateriale, samt at han er klar over sine rettigheder.

Rekruttering via annonce.

Når forsøgspersonerne kontakter afdelingen vil de pr. telefon blive orienteret om forsøgets karakter og indhold, de vil samtidig blive spurgt om oplysninger som kan sikre, de kan indgå i undrsøgelsen.

Hvis de fortsat er interesserede vil der derefter blive aftalt en tid for undersøgelse, og de vil samtidig får tilsendt relevant materiale, den skriftlige orientering og lægmandsorienteringen. Desuden vil pjecen ”Før du bestemmer dig” blive tilsendt.

Der vil samtidig blive aftalt en tid for en undersøgelse, hvor patientens samtykke endeligt kan blive indhentet og hvor det kan sikres at patienten har læst og forstået det skriftlige forsøgsmateriale, samt at han er klar over sine rettigheder.

Der vil ved de mundtlige orienteringer blive lagt vægt på

Undersøgelsesmetoder og forventelige ulemper for patienten

Formålet med undersøgelsen, og vigtigheden af det daglige indtag af broccolispirer.

Patienterne vil, inden de underskriver samtykket blive tilbudt en smagsprøve af broccolispirerne, så de ved, hvad der skal indtages og hvordan det smager.

### Datasikkerhed

Henvisning til fortrolige patientoplysninger via CPR-nummer og fuldt navn vil blive destrueret efter forsøgets afslutning. Forsøget vil blive registreret hos Datatilsynet.

### Financiering

Driftsudgifter betales fra en forskningsfond på Bispeberg Sygehus . Forskningsfonden består af overskud, som opstår når medicinalselskaber betaler udgifter til afprøvning af ny behandling på afdelingen. Ved ansøgningstidspunktet kommer sådan betaling fra 7 forskellige medicinal-selskaber, og disse selskaber har ingen interesser i forsøget. Fonden er underkastet offentlig revision.

Udgifter i forbindelse med analyse af blod og urin for indholdsstoffer fra broccolispirerne og disses metabolitter vil søges financieret via ansøgninger til diverse fonde støttende grundforskning, der vil i disse undersøgelser heller ikke forekomme nogen kommercielle interesser i forsøgene.

### Publikation

Resultater fra forsøget vil forsøges offentliggjort i et biomedicinsk tidsskrift med peer review.

### Referencer

1. Stocker, R. and J.F. Keaney, Jr., *Role of oxidative modifications in atherosclerosis.* Physiol Rev, 2004. **84**(4): p. 1381-478.

2. Griendling, K.K. and G.A. FitzGerald, *Oxidative stress and cardiovascular injury: Part II: animal and human studies.* Circulation, 2003. **108**(17): p. 2034-40.

3. London, S.J., et al., *Isothiocyanates, glutathione S-transferase M1 and T1 polymorphisms, and lung-cancer risk: a prospective study of men in Shanghai, China.* Lancet, 2000. **356**(9231): p. 724-9.

4. Zhu, C.Y. and S. Loft, *Effect of chemopreventive compounds from Brassica vegetables on NAD(P)H:quinone reductase and induction of DNA strand breaks in murine hepa1c1c7 cells.* Food Chem Toxicol, 2003. **41**(4): p. 455-62.

5. Fahey, J.W., Y. Zhang, and P. Talalay, *Broccoli sprouts: an exceptionally rich source of inducers of enzymes that protect against chemical carcinogens.* Proc Natl Acad Sci U S A, 1997. **94**(19): p. 10367-72.

6. Wu, L., et al., *Dietary approach to attenuate oxidative stress, hypertension, and inflammation in the cardiovascular system.* Proc Natl Acad Sci U S A, 2004. **101**(18): p. 7094-9.

7. Matusheski, N.V., J.A. Juvik, and E.H. Jeffery, *Heating decreases epithiospecifier protein activity and increases sulforaphane formation in broccoli.* Phytochemistry, 2004. **65**(9): p. 1273-81.

8. Matusheski, N.V., et al., *Preparative HPLC method for the purification of sulforaphane and sulforaphane nitrile from Brassica oleracea.* J Agric Food Chem, 2001. **49**(4): p. 1867-72.

9. Corretti, M.C., et al., *Guidelines for the ultrasound assessment of endothelial-dependent flow-mediated vasodilation of the brachial artery: a report of the International Brachial Artery Reactivity Task Force.* J Am Coll Cardiol, 2002. **39**(2): p. 257-65.

10. Shapiro, T.A., et al., *Chemoprotective glucosinolates and isothiocyanates of broccoli sprouts: metabolism and excretion in humans.* Cancer Epidemiol Biomarkers Prev, 2001. **10**(5): p. 501-8.

11. Lampe, J.W., et al., *Modulation of human glutathione S-transferases by botanically defined vegetable diets.* Cancer Epidemiol Biomarkers Prev, 2000. **9**(8): p. 787-93.
